# Supplementary material for: Rheumatoid Arthritis and CLOVES Syndrome: A Tricky Diagnosis
Source: Diagnostics (Basel). 2020 Jul 9;10(7):467. doi: 10.3390/diagnostics10070467 (PMC7400073; doi:10.3390/diagnostics10070467)
Supplement: Supplementary file 1 [file diagnostics-10-00467-s001.zip › Suppl/Suppl. Table 1.docx]

**Suppl. Table 1**: PI3k/AKT/mTOR pathway involvement in rheumatoid arthritis

| Type of process/cells | **Molecular mechanisms** | Ref. |
| --- | --- | --- |
| Macrophages | mTORC1 activates the NLRP3 inflammasome in macrophages | 3, 4 |
| T cells | PI3k/Akt/mTOR - negative regulator of Tregs, leading to expansion of proinflammatory Th1, Th17, Tfh; mTORC1 promotes Th17 differentiation | 4 |
| Fibroblast-like synovial cells | PI3Kδ regulates PDGF-mediated FLS growth, survival, migration and resistance to apoptosis  PTEN- negative pathway regulator- reduced in FLS | 1, 6 |
| Osteoclasts | mTOR1 activity increased in osteoclasts  mTOR inhibition downregulates extracellular matrix catabolic enzymes | 4, 6 |
| Chondrocytes | mTOR1 controls chondrocyte survival, activated by mechanical stress  PI3k/AKT/mTOR signaling contributes to inflammatory cartilage damage | 4 ,6 |
| Cytokines metabolism | M-CSF and RANKL activate PI3K and AKT  TNFα activates mTOR | 3, 4, 6 |

**Legend:** AKT - protein-kinase B, FLS- fibroblast-like synovial cells, mTOR- mammalian target of rapamycin, PDGF-platelet-derived growth factor, PI3K -phosphatidylinositol 3, 4, 5 triphosphate kinase, PTEN -phosphatase and tensin homolog, RA-rheumatoid arthritis, Th1- T helper 1, Th17-T helper 17, Tfh –T follicular helper, Tregs - T regulatory cells, M-CSF –macrophage colony stimulating factor, RANKL - receptor activator of nuclear kappa-B ligand.

**Suppl. Fig. 1**

MRI of the right hand, MERGE sequence, coronal view demonstrates elongated 2^nd^ and 3^rd^ finger (white arrows) associated with slight ulnar deviation of the third finger.

**Suppl. Fig. 2**

MRI of the right hand, STIR sequence, axial view demonstrates mild fluid collection at the level of the first carpometacarpal joint space (*white arrow*).
