# Supplementary figures and images for: Rheumatoid Arthritis and CLOVES Syndrome: A Tricky Diagnosis
Source: Diagnostics (Basel). 2020 Jul 9;10(7):467. doi: 10.3390/diagnostics10070467 (PMC7400073; doi:10.3390/diagnostics10070467)

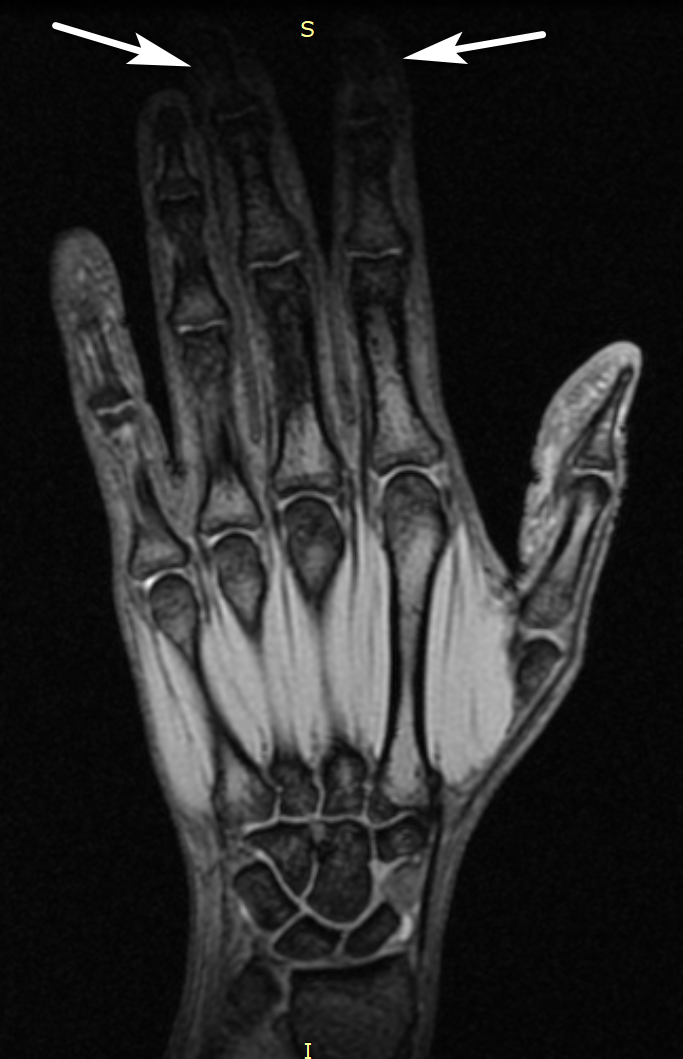

Supplement: Supplementary file 1 [file diagnostics-10-00467-s001.zip › Suppl/Suppl. Fig 1.tif]

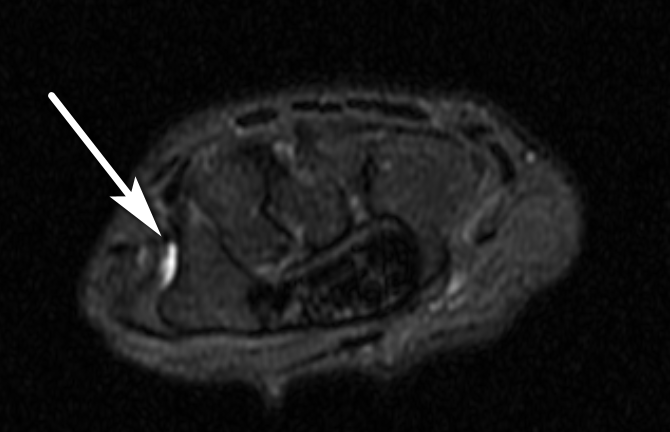

Supplement: Supplementary file 1 [file diagnostics-10-00467-s001.zip › Suppl/Suppl. Fig 2.tif]
